# Static and Dynamic Disorder in Formamidinium Lead Bromide Single Crystals

**DOI:** 10.1021/acs.jpclett.2c03337

**Published:** 2023-02-01

**Authors:** Guy Reuveni, Yael Diskin-Posner, Christian Gehrmann, Shravan Godse, Giannis G. Gkikas, Isaac Buchine, Sigalit Aharon, Roman Korobko, Constantinos C. Stoumpos, David A. Egger, Omer Yaffe

**Affiliations:** † Department of Chemical and Biological Physics, Weizmann Institute of Science, Rehovot76100, Israel; ‡ Chemical Research Support, Weizmann Institute of Science, Rehovot76100, Israel; § Department of Physics, Technical University of Munich, 85748Garching, Germany; ∥ Department of Materials Science and Technology, University of Crete, Voutes Campus, Heraklion, GR70013, Greece; ⊥ Department of Chemistry and Institute of Nanotechnology and Advanced Materials, Bar-Ilan University, Ramat Gan5290002, Israel

## Abstract

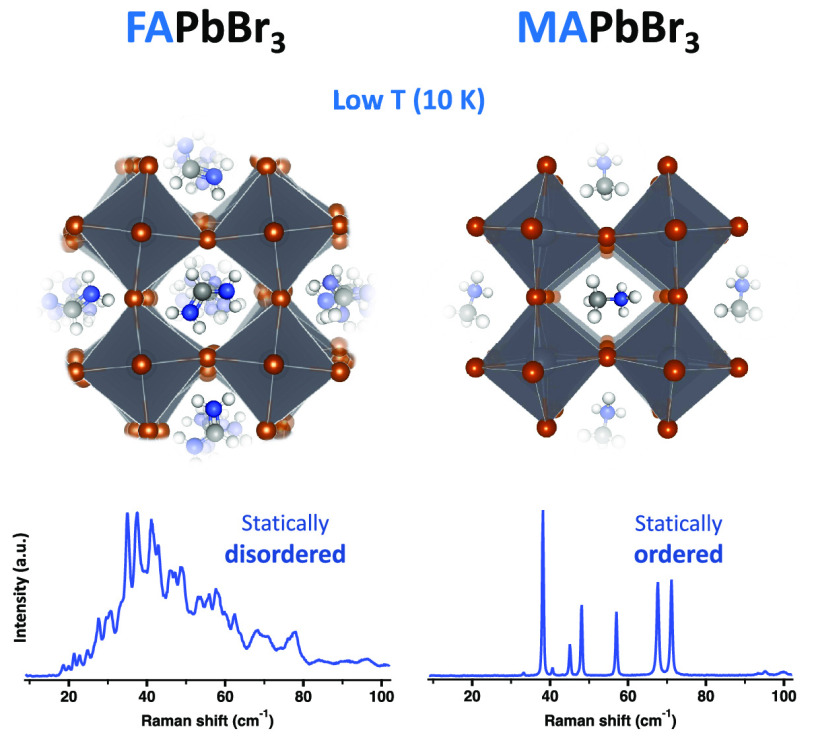

We show that formamidinium-based crystals are distinct from methylammonium-based halide perovskite crystals because their inorganic sublattice exhibits intrinsic local static disorder that coexists with a well-defined average crystal structure. Our study combines terahertz-range Raman scattering with single-crystal X-ray diffraction and first-principles calculations to probe the evolution of inorganic sublattice dynamics with temperature in the range of 10–300 K. The temperature evolution of the Raman spectra shows that low-temperature, local static disorder strongly affects the crystal structural dynamics and phase transitions at higher temperatures.

Extensive research on lead halide perovskites (APbX_3_, where X = Cl, Br, or I) has been primarily motivated by potential photovoltaic applications.^1−12^ It has challenged the use of the primitive unit cell, together with the average crystal structure as determined by X-ray diffraction (XRD) measurements it represents for explaining certain properties of these crystals. Specifically, we and others have shown that at sufficiently high temperatures, halide perovskites exhibit large-amplitude (i.e., anharmonic) PbX_6_ octahedral rotations and distortions.^13−19^ Strongly anharmonic dynamic disorder implies that the actual crystal structure includes local motifs that are not captured when the material is represented by its average structure using the primitive unit cell.^20,21^ Notably, these local structural motifs were shown to impact the optoelectronic properties of lead halide perovskites in a profound manner.^22−26^

When the effect of dynamic disorder is negligible at sufficiently low temperatures, lead halide perovskites based on the methylammonium (MA) cation do not exhibit disorder.^27−30^ Therefore, the representation of these crystals by a primitive unit cell can adequately describe their properties. By contrast, recent studies of lead halide perovskites with formamidinium (FA) as an A-site cation demonstrated a high degree of orientational disorder of the FA cation within the inorganic framework, even at cryogenic temperatures.^13,31−34^ Because the FA cation is large and disordered and interacts with the lead and the X-site ions electrostatically,^35^ it is likely to distort the perovskite inorganic framework.^36,37^ At higher temperatures that are more relevant for device-operating conditions, the interplay of the orientational, static disorder of FA with the dynamic disorder in lead halide perovskites may impact their optoelectronic properties but remains mostly unexplored to the best of our knowledge.

In this work, we investigate the possibility that varying amounts of static and dynamic disorder coexist in FA-based lead halide perovskites by studying how the inorganic sublattice dynamics of FAPbBr_3_ differ from those of its MA-based counterpart MAPbBr_3_. We combine terahertz-range Raman scattering with single-crystal X-ray diffraction (scXRD) and first-principles calculations to probe the structural dynamics of the PbBr_6_ framework in FAPbBr_3_ as well as its temperature evolution (10–300 K) and compare it to the well-known case of MAPbBr_3_. We show that contrary to MAPbBr_3_, the PbBr_6_ framework in FAPbBr_3_ single crystals exhibits a significant degree of local static disorder despite having a well-defined average structure. Our findings suggest that the static disorder at low temperatures is related to the bulky FA molecule and demonstrate that it augments the dynamic disorder present at higher temperatures in FAPbBr_3_, which potentially has significant implications for the optoelectronic and thermal stability properties of FA-based lead halide perovskites.

FAPbBr_3_ and MAPbBr_3_ crystals were grown via the inverse temperature crystallization method and the antisolvent method, respectively.^38,39^ Details regarding crystal growth, measurement apparatus, and parameters are given in the Supporting Information. Figure 1a presents a comparison between the unpolarized (i.e., summing over all polarization angles) terahertz-range Raman spectra of FAPbBr_3_ (red) and MAPbBr_3_ (black) single crystals at 10 K. The features in the Raman spectrum of MAPbBr_3_ are well-resolved and agree with factor group analysis predictions based on the average crystal structure.^17,40,41^ In sharp contrast, the Raman spectrum of FAPbBr_3_ exhibits a strong background and contains more than 40 sharp peaks, higher than the expected number of Raman-active lattice modes (∼18, depending on the specific space group and cell size used in factor group analysis^42^). These striking differences between the two spectra indicate that FAPbBr_3_ is showing a high degree of static disorder of unknown origin, which adds to recent discussions on the pertinent mechanisms underlying the interplay of static and dynamic disorder in lead halide perovskites.^43−45^

**Figure 1:**
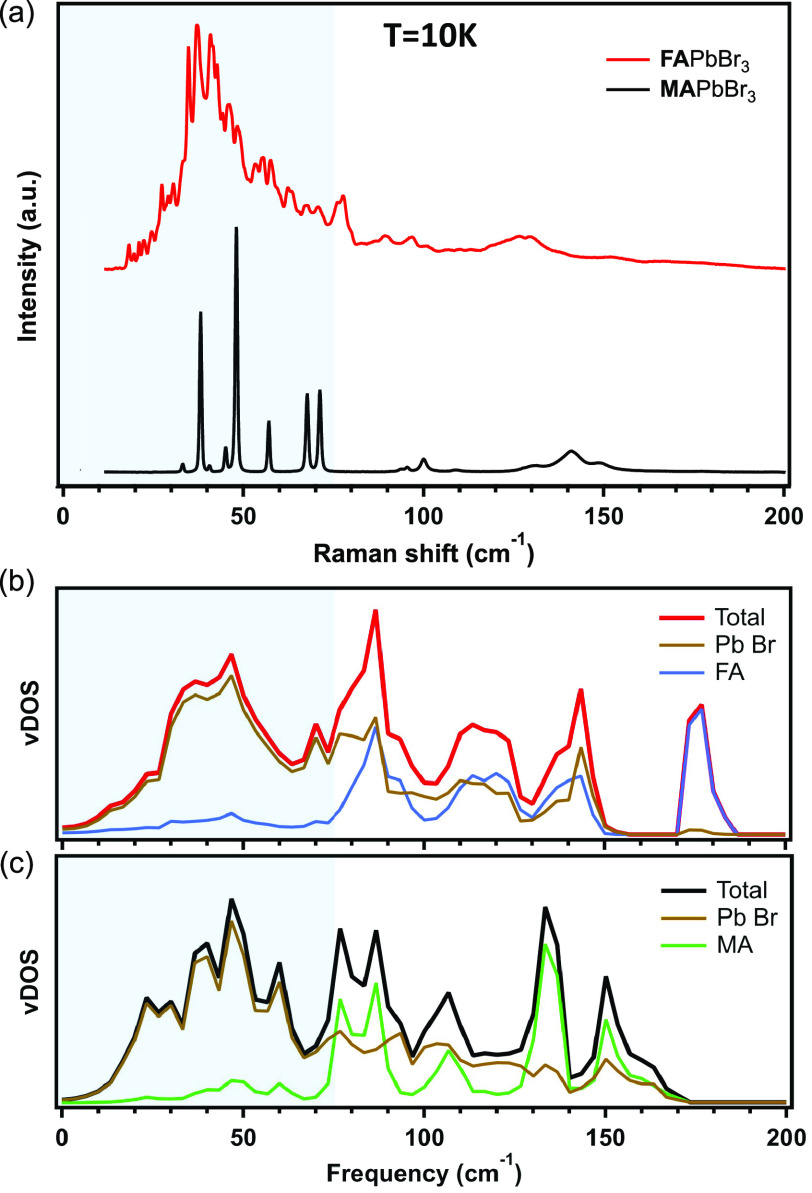
(a) Raman-scattering spectra of FAPbBr_3_ (red) and MAPbBr_3_ (black) single crystals at 10 K. The spectra are offset for the sake of clarity. Vibrational density of states (vDOS) as well as decomposition of the vDOS for the organic and inorganic sublattices of (b) FAPbBr_3_ and (c) MAPbBr_3_, as calculated by density functional theory. The gray-shaded area marks the frequency range in which the vDOS predominantly features vibrations of the PbBr_6_ framework rather than the organic cation. Note that the vDOS also includes contributions from Raman-inactive modes and that it is shown for a selected frequency region that does not, e.g., show contributions from imaginary modes and higher-frequency molecular modes.

One possible explanation for a large number of Raman peaks is that the relatively large FA cation^46,47^ may introduce additional modes into the terahertz frequency range. Molecular modes are not accounted for in factor group analysis, which considers only the space group of the average crystal (i.e., the organic cation is treated as a sphere). To examine if the FA cation does indeed significantly change the low-frequency vibrational features in FAPbBr_3_, we compute the vibrational density of states (vDOS) of cubic FAPbBr_3_ and MAPbBr_3_ using density functional theory (DFT) employing the VASP code.^48^ We calculated the vDOS of the cubic structures because, for FAPbBr_3_, only the cubic phase was stable enough to perform phonon calculations (see the Supporting Information for further details). The total and decomposed (Pb–Br framework and organic cation) vDOS of FAPbBr_3_ and MAPbBr_3_ are presented in panels b and c, respectively, of Figure 1. The comparison finds the overall appearance of the spectral features to be rather similar, with the exception that the vibrations associated with the rigid-body motion of the molecules, in the range between ∼125 and ∼180 cm^–1^, exhibit some differences, in line with the different moments of inertia and molecular masses of FA and MA and previous findings reported for MAPbI_3_ and FAPbI_3_.^49^ Importantly, the results show that in FAPbBr_3_ the low-frequency part of the vDOS up to approximately 75 cm^–1^ (shaded area), i.e., the region that contains a multitude of sharp Raman features in the experiments (cf. Figure 1a), predominantly stems from vibrations of the inorganic PbBr_6_ framework. Together with the similarity of the computed vDOS of FAPbBr_3_ and MAPbBr_3_ in this low-frequency region, it suggests that the large number of peaks observed in the Raman spectrum of FAPbBr_3_ at 10 K is rooted in significant distortions of the inorganic framework, which are absent in our phonon calculations of the cubic structure. We note in passing that the identification of the origin of the static disorder in FAPbBr_3_ by harmonic phonon calculations is not expected to be modified by anharmonic effects, which in these materials do not strongly alter the atomic contributions to the vDOS.^50^

To test if the multitude of peaks in the Raman spectra of FAPbBr_3_ at 10 K results from nanodomains that form during the cooling process, we performed polarization–orientation (PO) Raman measurements at 10 K (Figure 2a). We measure the change in Raman-scattering intensity as a function of the angle between the linear polarization of the excitation laser and an arbitrary axis in the plane of the crystal surface (Figure S4). This is useful because the fluctuations in intensity as a function of the polarization angle reflect the average symmetry of the measured sample.^14,51^ Despite exhibiting a broad spectrum that does not obey the expected Raman selection rules, FAPbBr_3_ evidently also features a periodic PO Raman dependence, similar to that of MAPbBr_3_ (Figure S5). These results indicate that our FAPbBr_3_ crystal was not fractured into nanodomains because the PO dependence of a multidomain system would not exhibit any periodicity. Therefore, the data presented in Figure 2a highlight an inherent confluence of properties in FAPbBr_3_, demonstrating local disorder as expressed in the unpolarized Raman spectrum while still being crystalline on average.

**Figure 2:**
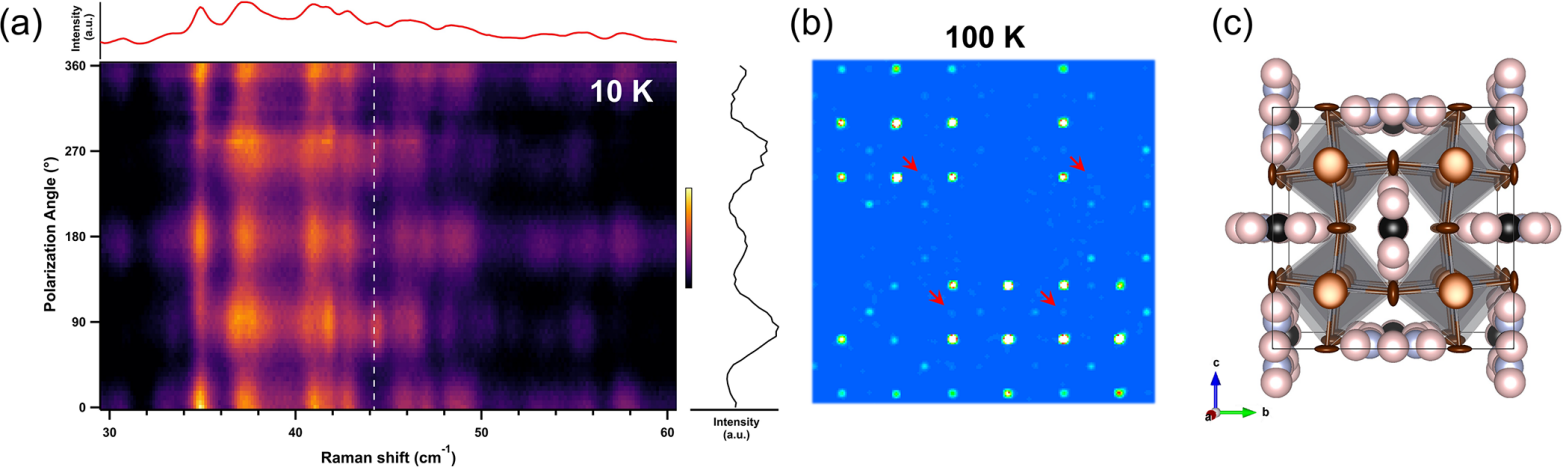
FAPbBr_3_ crystallography through polarization–orientation Raman scattering and single-crystal X-ray diffraction. (a) False-color polarization–orientation Raman plot of FAPbBr_3_ at 10 K. The top panel shows the unpolarized Raman spectrum (as depicted in Figure 1a), and the right panel shows the cross section of the 44 cm^–1^ peak (marked by a white dashed line), presenting its polarization-dependent intensity. Spectra were normalized to the highest peak; their intensities are represented by the color scale. (b) Precession image of FAPbBr_3_ at 100 K [*Immm* space group, (10–1) projection]. Red arrows point at some non-indexed satellite reflections of very low intensity, corresponding to a larger (likely doubled) supercell. Bright reflections correspond to the indexed reflections of the *Immm* space group (#71). (c) Schematic representation of the 100 K crystal structure of FAPbBr_3_ obtained from single-crystal X-ray diffraction measurements. Gray, brown, blue, black, and pink spheres denote Pb, Br, N, C, and H atoms, respectively.

To further investigate the crystallinity of our sample, we conducted scXRD measurements on the same FAPbBr_3_ crystal at 100 K. The comparison to the Raman data at 10 K is valid because, for this material, there are no reported phase transitions below 100 K.^52−54^Figure 2b shows the precession image from scXRD at 100 K, where the reflection pattern appears as ordered, confirming that the inorganic PbBr_6_ framework exhibits a well-defined average structure. Some low-intensity, non-indexed satellite reflections (marked with red arrows) appeared only in the 100 K data but not at any of the higher temperatures also measured in scXRD (see section S2 of the Supporting Information), suggesting the emergence of a supercell with doubled dimensions with respect to the *Immm* unit cell. The weak intensity of the satellite peaks precludes meaningful data integration regarding the supercell settings. Specifically, while the refinement of the reflection pattern at 100 K showed a slight preference for the orthorhombic *Immm* space group over other space groups that were identified by group theoretical analysis,^55−57^ other independent structural refinements are ambiguous (see the Supporting Information for details). The ambiguity of the refinement, in addition to the Raman data shown in Figure 1a, indicates the presence of local disorder within the inorganic sublattice. Altogether, the presence of these local disorder domains leads to an average structure of various local symmetries, each of which describes the XRD reflection pattern in an essentially equivalent manner.

Finally, we compare the temperature evolution of the structural dynamics of both lead halide perovskite crystals. Figure 3a shows a false-color map representing the temperature-dependent Raman-scattering spectra of FAPbBr_3_ (left) and MAPbBr_3_ (right). As the temperature increases, the continuous broadening and red-shifting of the Raman features are apparent in both materials. The data show that MAPbBr_3_ exhibits an order–disorder phase transition from orthorhombic to tetragonal around 145 K, similar to previous reports.^28,58^ This phase transition is noticeable as a substantial broadening of the spectrum due to strongly anharmonic thermal fluctuations of the PbBr_6_ octahedra,^17,41^ i.e., due to dynamic disorder. FAPbBr_3_ shows a similar temperature evolution with apparent phase transitions at similar temperatures, with two noticeable spectral changes occurring around 115 and 155 K (marked with white arrows), similar to the reported temperatures of structural phase transformations in FAPbBr_3_.^54^ However, these spectral changes are mild compared to what is observed in MAPbBr_3_. These differences are due to the fact that the Raman spectrum of FAPbBr_3_ is already very broad at low temperatures, as discussed above, to the extent that a phase transition does not result in further drastic changes. Nonetheless, as the temperature increases, dynamic disorder gradually becomes more significant also in this case, and accordingly, the Raman spectra of FAPbBr_3_ and MAPbBr_3_ become more alike. In Figure 3b, we compare the Raman spectra of FAPbBr_3_ and MAPbBr_3_ at 300 K. At this temperature, both spectra are dominated by dynamic disorder and exhibit diffused intensity at low frequency (<50 cm^–1^). The main difference between the spectra occurs between 50 and 170 cm^–1^ (shaded area), where the relative intensity is significantly higher for FAPbBr_3_. We hypothesize that this increased intensity indicates that the motions of the FA molecule in the inorganic cage distort the PbBr_6_ framework while that of the MA molecule does not. The notion is relevant for the mechanistic understanding of the optoelectronic properties in FA-based lead halide perovskites because the electronic states due to the PbBr_6_ framework are close to the band edges. In light of this and the improved stability and self-healing properties of FA-based materials,^39,59,60^ it is worth investigating the role of the FA molecule for the dynamic disorder of the PbBr_6_ framework in future experimental and theoretical work.

**Figure 3:**
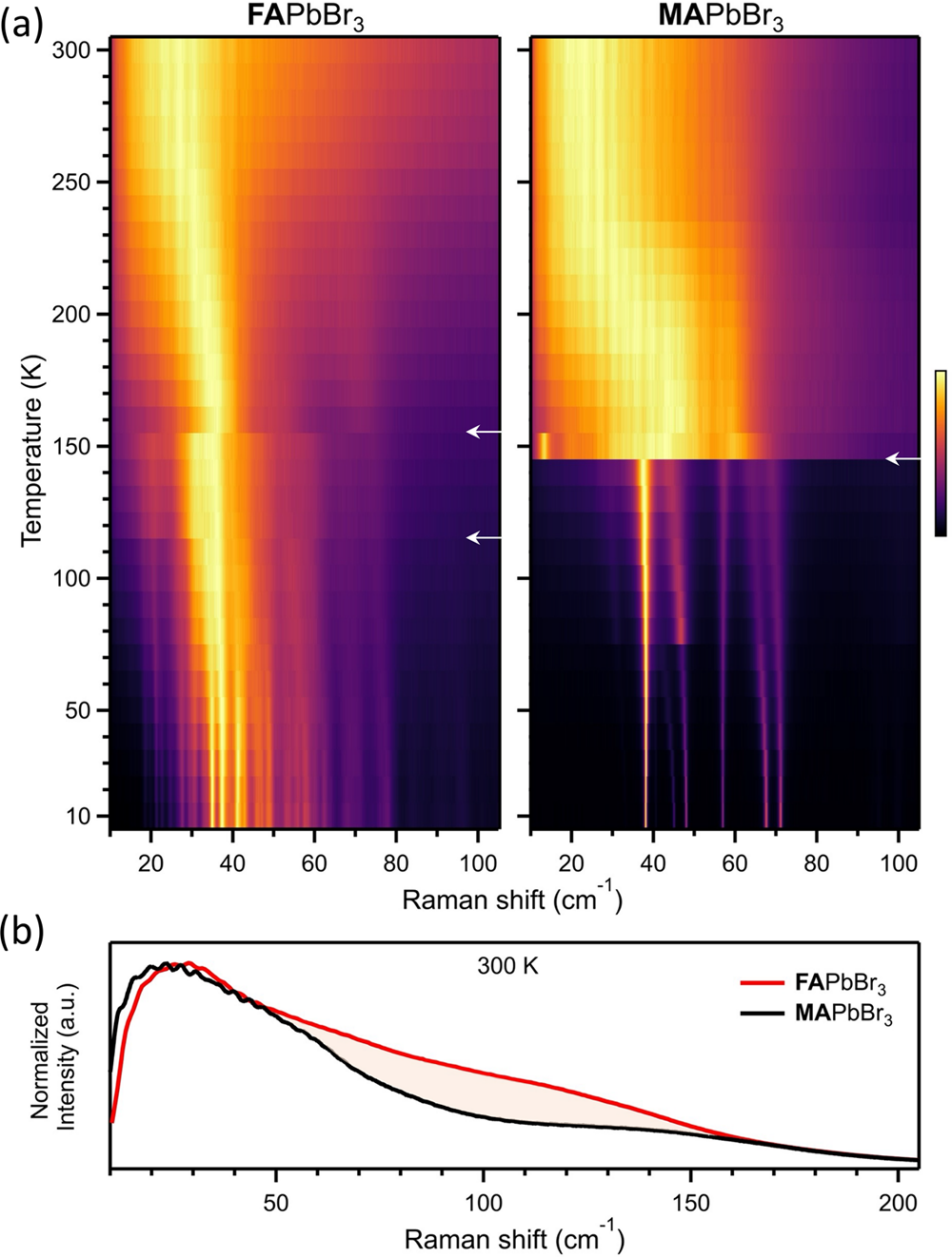
(a) Temperature-dependent Raman spectra from 10 to 300 K, measured at 10 K intervals, of FAPbBr_3_ (left) and MAPbBr_3_ (right). All spectra were normalized to the highest peak, represented by brighter colors according to the color scale. White arrows point to observed phase transitions. (b) Raman spectra of the two crystals at 300 K. The shaded area highlights the intensity difference.

In conclusion, we used terahertz-range Raman scattering, single-crystal X-ray diffraction, and first-principles calculations to show that contrary to MAPbBr_3_, the PbBr_6_ framework in FAPbBr_3_ is intrinsically disordered while having a well-defined average structure at cryogenic temperatures. Consequently, the local structure does not coincide with the average structure, and a supercell accounting for the FA cation orientational disorder, together with the resulting distortions of the inorganic framework, may better describe the physical state of the material. When the temperature increases, the dynamic disorder becomes more significant, leading to a high degree of resemblance between MAPbBr_3_ and FAPbBr_3_, distinct only by the higher degree of static disorder of the latter. FAPbBr_3_ thus serves as an intriguing system that combines long-range crystal order with inherent local, static disorder, potentially being a key to describing the complex behavior of this remarkable perovskite and guiding future studies of related FA-based perovskites.

## Supporting Information Available

Crystal structures of FAPbBr_3_ from single-crystal XRD through traditional refinement at 300 K (*Pm*3̅*m*), 250 K (*Pm*3̅*m*), 200 K (*Pm*3̅*m*), 150 K (*Pm*3̅*m*), and 100 K (*Pm*3̅*m*), and through group–subgroup refinement at 100 K (*Immm*, *Pbnm*, and *Cmcm*) (ZIP)
Crystal synthesis procedures, discussion of X-ray diffraction and refinement processes, structural data, additional polarization-dependent Raman measurements in various temperatures, and details of the experimental and computational methods (PDF)
Transparent Peer Review report available (PDF)
