# Supplementary material for: Static and Dynamic Disorder in Formamidinium Lead Bromide Single Crystals
Source: J Phys Chem Lett. 2023 Feb 1;14(5):1288–93. doi: 10.1021/acs.jpclett.2c03337 (PMC9923750; doi:10.1021/acs.jpclett.2c03337)
Supplement: Supplementary file 2 — jz2c03337_si_002.pdf [file jz2c03337_si_002.pdf]

Supporting Information

Static and Dynamic Disorder in Formamidinium Lead Bromide Single Crystals

Guy Reuveni,[1] Yael Diskin-Posner,[2] Christian Gehrmann,[3] Shravan Godse,[3] Giannis G. Gkikas,[4] Isaac Buchine,[5] Sigalit Aharon,[1] Roman Korobko,[1] Constantinos C. Stoumpos,[4] David A. Egger,[3] and Omer Yaffe[1,*]

[1]*Department of Chemical and Biological Physics, Weizmann Institute of Science, Rehovot 76100, Israel;*

[2]*Chemical Research Support, Weizmann Institute of Science, Rehovot 76100, Israel;*

[3]*Department of Physics, Technical University of Munich, 85748 Garching, Germany;*

[4]*Department of Materials Science and Technology, University of Crete, Voutes Campus, Heraklion GR-70013, Greece;*

[5]*Department of Chemistry and Institute of Nanotechnology and Advanced Materials, Bar-Ilan University, Ramat Gan 5290002, Israel;*

[*] omer.yaffe@weizmann.ac.il

S1. CRYSTAL SYNTHESIS PROCEDURES

S1.a. $FAPbBr_3$

$FAPbBr_3$ was crystalized in the inverse temperature crystallization method to induce high quality crystallization.[1] FABr (1.1M) and $PbBr_2$ (1.0M) were mixed in a 1:1 ratio of γ-Butyrolactone (GBL) and Dimethylformamide (DMF). The precursor solution is stirred for 2 hours at room temperature and is then filtered directly into a crystallization plate. The plate is tightly covered with aluminum foil and placed into a pre-heated oven at 50ºC. The oven's temperature is then ramped slowly up to 80ºC over 5 hours. Upon crystallization, the plate is removed, and crystals are dried quickly to prevent them from dissolving as a result of ambient temperature.

S1.b. $MAPbBr_3$

$MAPbBr_3$ crystals were synthesized by the antisolvent method.[2] MABr (Dyesol) and $PbBr_2$ (Dyesol) were dissolved in DMF (N,N-dimethylformamide (Aldrich)) at the same time to obtain a 0.88 M solution in MA^+ and a 0.80 M solution in Pb^{2+} (mixing at room temperature and in ambient air (RH 45%)). It is important to dissolve both chemicals in the same liquid, because the solubility of $PbBr_2$ is increased by the presence of MABr. The solution was then filtered and put in an open vial. The open vial was placed in a wider bottle, containing ethyl acetate (Bio-Lab) in excess. The DMF solution, initially occupying half the available volume of the vial, absorbs ethyl acetate, which is an antisolvent for the halide perovskite. Crystals started to form and grew till the level of the DMF- ethyl acetate-enriched solution occupied all the available volume in the vial (between 24 h and 48 h in our case). The crystals were then extracted from the solution, carefully dried with blotting paper, washed with ethyl acetate and dried again.

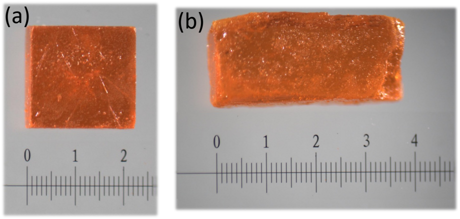

FIG. S1: Microscope images of (a) $FAPbBr_3$ and (b) $MAPbBr_3$ single crystals. The images were taken a long time after the measurements, thus they are not transparent as fresh crystals. Scale marks are in millimeter.

S2. X-RAY DIFFRACTION

The crystal structure and symmetry of the $FAPbBr_3$ was determined by single-crystal X-ray diffraction (scXRD). The data and the refined structures can be found in the CIFs attached to the SI. Albeit slightly higher refinement parameters resulted from an orthorhombic *Immm* (#74) space group, all space groups that are presented here have similarly described the reflection patterns. This ambiguity suggests a high lattice disorder that leads to an average structure combining various crystal symmetries.

S2.a. Measurement procedure

The synthesized $FAPbBr_3$ crystal was broken to small pieces, then immersed in a small amount of nail polish and mounted onto a broken Mitogen loop, at room temperature (see fig. S2). Measurements were performed on a $FAPbBr_3$ single crystal starting at 300 K, then the crystal was cooled down to 100 K at a rate of 1 K/min. The cooling descend paused at 250 K, 200 K, 150 K for a data collection. A 5 minutes wait before starting each measurement allowed the crystal to completely adjust to the temperature. All data were collected on Rigaku Synergy-S dual source diffractometer equipped with Dectris Pilatus3 R CdTe 300 K detector and microfocus with AgKα (λ=0.56087 Å), with ω scans. This specific wavelength and detector setting is the most equipped in measuring these materials today. Data were collected to a very high resolution and redundancy. We have performed two independent structural solution and refinement processes. These two independent refinement solutions are presented herein.

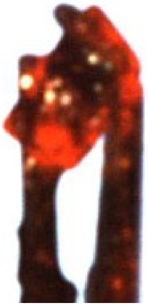

FIG. S2: The mounted $FAPbBr_3$ crystal.

S2.b. Traditional refinement

The traditional data processing found no systematic absentees in the diffraction pattern (see table S1) and therefore with lack of any other information we set the symmetry to *Pm-3m*, where structure is solved and refined to a very low R factor. Data were integrated and processed according to the highest and most suitable Laue symmetry with CrysAlis^{PRO} and a Gaussian absorption correction was applied. Structures were solved using SHELXT[3]

and further refined with SHELXL[4] by full matrix least-squares refinement based on F^2. The major contributors to the diffraction scattering are the Pb and Br atoms and their position is quickly determined. The lead bromide sublattice, which is composed of very good contributors to the diffraction scattering pattern, defines the crystal symmetry. The formamidinium molecule in the cavity is probably disordered and has a negligible contribution to the diffraction scattering pattern. Its atoms' contribution to the diffraction scattering pattern is extremely small, even at low temperatures as 100 K. A small undefined electron density blob exists in the electron density map, which is contributed by one disordered formamidinium molecule sitting on a symmetry fold, even with data sets measured to a very high resolution such as 0.4 Å. Therefore, the symmetry and space group are defined exclusively by the diffraction scattering from the lead and bromine atoms.

When temperature is decreasing bellow 150 K, the bromine atom is shifting away from its special position and creating a conformation change in the lattice (see table S2). Since the bromine atom's contribution to the diffraction pattern is substantial, the shift from the special position is genuine and followed by a dramatic decrease in the R factor during the refinement. The shift in the bromine atom position between the 150 K to the 100 K measurements is changing the lattice conformation, which might be indication of a phase transition. The refinement was completed by refining a formamidinium fragment into the remaining electron density. The fragment belonged to the TALTAE structure from the CCDC.

S2.c. Group-subgroup based refinement

The second set of independent structural refinement is focused on the three main space groups indicated by group theoretical analysis,[5–7] at 100 K. Crystallographic data specific for the 100 K data set are summarized in tables S3- S7.[8,9] Raw data were indexed with a variety of possible supercells of the primitive $P4/mbm$ space group, that produced reasonably good indexing of the observed reflections (>90%). Each indexing attempt was accompanied by a separate integration process (CrysAlis^{PRO} platform) resulting in different sets of hkl files that were subsequently used to solve the structure (Superflip, integrated in Jana2006) and subsequently refine it (Jana2006). We observe a good agreement with all three possible orthorhombic space groups deriving from the tetragonal β-phase ($P4/mbm$ space group),

namely *Pbnm*, *Cmcm*, and *Immm*, and find a preferable agreement with the latter. Figure S3 presents precession images for 300 K, 200 K and 100 K, demonstrating the ordered reflection pattern, approving our sample's crystallinity.

Fig. 2c in the main article portrays the orthorhombic *Immm* structure, which demonstrates through thermal ellipsoids how the bromine atoms (brown spheres) are highly shifted from their special Wyckoff position, compared to other atoms in the structure. Some non-indexed satellite reflections (red circles in fig. S3(c)) appeared only in the 100 K data, which may suggest the emergence of a large supercell. The approach of refining the structure as a supercell is a different, yet reasonable approach that further implies the existence of inherent disorder. That is because a supercell can include many different cation orientations

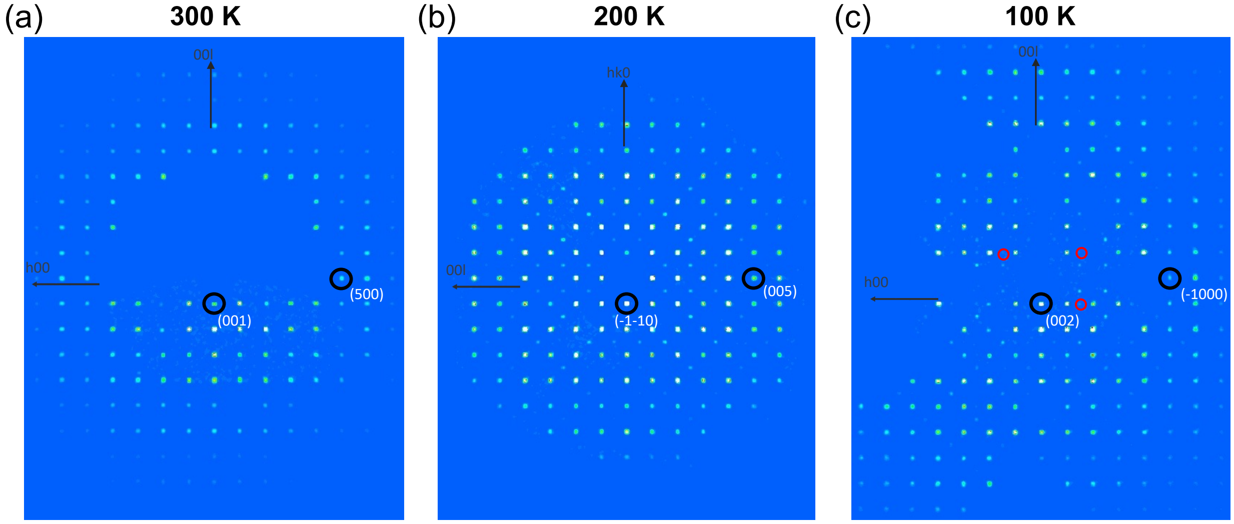

FIG. S3: Precession images of $FAPbBr_3$ at (a) 300K (*Pm-3m* space group, (10-1) projection), (b) 200K (*P4/mbm* space group, (221) projection), and (c) 100K (*Immm* space group, (10-1) projection). Red circles represent non-indexed satellite reflections of very low intensity, suggesting the emergence of a large supercell.

alongside the inorganic framework distortions. This suggests that the inorganic framework of $FAPbBr_3$ deforms in a very different manner compared to that of $MAPbBr_3$, possibly driven by the planar nature of the FA cation which tends to orient in discrete directions along the three orthogonal axes, in contrast to MA which tends to orient along the body diagonals of the cage.[10]

TABLE S1: Temperature dependent crystal data and structure refinement for $FAPbBr_3$ - 1st independent refinement

Temperature	300 K	250 K	200 K	150 K	100 K
Chemical Formula			$HC(NH_2)_2PbBr_3$		
Formula weight			492		
Crystal system	cubic	cubic	cubic	cubic	cubic
Space group	*Pm-3m*	*Pm-3m*	*Pm-3m*	*Pm-3m*	*Pm-3m*
a (Å)	5.98010(10)	5.97200(10)	5.95470(10)	5.93280(10)	5.91992(4)
Volume (Å^3)	213.858(11)	212.990(11)	211.145(11)	209.140(11)	207.466(4)
Z	1	1	1	1	1
Calculated Density (g/cm^3)	3.820	3.836	3.869	3.906	3.938
μ (mm^{-1})	18.134	18.208	18.367	18.543	18.693
Index ranges	$-12\leq h \leq 12$<br>$-12\leq k \leq 12$<br>$-12\leq l \leq 12$	$-7\leq h \leq 7$<br>$-7\leq k \leq 7$<br>$-7\leq l \leq 7$	$-7\leq h \leq 7$<br>$-7\leq k \leq 7$<br>$-7\leq l \leq 7$	$-7\leq h \leq 7$<br>$-7\leq k \leq 7$<br>$-7\leq l \leq 7$	$-12\leq h \leq 12$<br>$-12\leq k \leq 12$<br>$-12\leq l \leq 12$
No. of reflection (unique)	24055(243)	5236 (78)	5198 (78)	5176 (76)	23410 (233)
$\theta_{max}(^{\circ})$	35.053	21.700	21.766	21.654	34.917
R_{int}	0.0418	0.0761	0.0712	0.833	0.0659
Completeness to θ (%)	1.000	1.000	1.000	1.000	1.000
Data / restraints / parameters	243 / 12 / 25	78 / 3 / 13	78 / 3 / 13	76 / 3 / 13	233 / 12 / 26
Goodness-of-fit on F^2	1.093	1.277	1.372	1.298	1.296
Final R_1 and wR_2 indices [I> 2σ(I)]	0.0145, 0.0336	0.0184, 0.0581	0.0291, 0.0848	0.0262, 0.0815	0.0086, 0.0176
R_1 and wR_2 indices [all data]	0.0159, 0.0339	0.0184, 0.0581	0.0291, 0.0848	0.0262, 0.0815	0.0086, 0.0176

TABLE S2: Structure parameters - 1st independent refinement

Temperature	300 K	250 K	200 K	150 K	100 K
Pb(1) - Br(1) Bond length (Å)	2.99005(5)	2.98600(5)	2.97735(5)	2.96790(5)	2.97671(16)
Pb(1) (x,y,z)	0.500000<br>0.500000<br>0.500000	0.500000<br>0.500000<br>0.500000	0.500000<br>0.500000<br>0.500000	0.500000<br>0.500000<br>0.500000	0.500000<br>0.500000<br>0.500000
Br(1) (x,y,z)	0.000000<br>0.500000<br>0.500000	0.000000<br>0.500000<br>0.500000	0.000000<br>0.500000<br>0.500000	0.000000<br>0.500000<br>0.500000	0.4467(2)<br>0.500000<br>0.000000

TABLE S3: Crystal data and structure refinement for $FAPbBr_3$ at 100 K - 2nd independent refinement

Chemical Formula		$HC(NH_2)_2PbBr_3$	
Formula weight		492	
Temperature		100 K	
Wavelength		0.56087 Å	
Crystal system		Orthorhombic	
Space group	*Immm*	*Pbnm*	*Cmcm*
Unit cell dimensions	a=11.8423(3) Å, α=90° b=11.8411(3) Å, β=90° c=11.8346(3) Å, β=90°	a=8.3673(2) Å, α=90° b=8.3759(2) Å, β=90° c=11.8445(3) Å, β=90°	a=11.8432(3) Å, α=90° b=11.8347(3) Å, β=90° c=11.8406(3) Å, β=90°
Volume (Å^3)	1659.52(7)	830.11(4)	1659.59(7)
Z	8	4	8
Calculated Density (g/cm^3)	3.9382	3.9365	3.938
Absorption coefficient (mm^{-1})	18.793	18.785	18.792
F(000)	1696	848	1696
Crystal size (mm)	0.143x0.089x0.023	0.143x0.089x0.023	0.143x0.089x0.023
θ range for data collection	2.71 to 35°	2.35 to 35.21°	2.35 to 35.23°
Index ranges	$-22\leq h \leq 24$, $-23\leq k \leq 21$, $-24\leq l \leq 21$	$-16\leq h \leq 16$, $-17\leq k \leq 14$, $-24\leq l \leq 22$	$-22\leq h \leq 24$, $-24\leq k \leq 22$, $-23\leq l \leq 23$
Reflections collected	23768	22648	23681
Independent reflections	3960 [R_{int} = 0.0502]	3612 [R_{int} = 0.0511]	3959 [R_{int} = 0.0506]
Completeness	98% (to θ = 34.85°)	98% (to θ = 23.89°)	98% (to θ = 34.85°)
Refinement method		Full-matrix least-squares on F^2	
Data / restraints / parameters	3960 / 4 / 39	3612 / 2 / 28	3959 / 2 / 36
Goodness-of-fit	1.63	2.50	2.68
Final R indices [I> 2σ(I)]	R_{obs}=0.0425, wR_{obs}=0.0914	R_{obs}=0.0642, wR_{obs}=0.1492	R_{obs}=0.0660, wR_{obs}=0.1578
R indices [all data]	R_{all} = 0.0763, wR_{all} = 0.0990	R_{all} = 0.1052, wR_{all} = 0.1588	R_{all} = 0.1045, wR_{all} = 0.1665
Domain fractions	#1: 27.6(4)% (1 0 0 0 1 0 0 0 1) #2: 38.8(2)% (0 0 1 0 -1 0 1 0 0) #3: 0.335(3) (0 1 0 1 0 0 0 0 -1)	#1: 100% (1 0 0 0 1 0 0 0 1)	#1: 55.9(5)% (1 0 0 0 1 0 0 0 1) #2: 44.1(5)% (0 -1 0 -1 0 0 0 0 -1)
Largest diff. peak and hole	10.27 and -6.78 e·Å^{-3}	7.55 and -6.68 e·Å^{-3}	7.69 and -11.39 e·Å^{-3}

$$R = \frac{\Sigma||F_o|-|F_c||}{\Sigma|F_o|},\ wR = \left(\frac{\Sigma[w(|F_o|^2-|F_c|^2)^2]}{\Sigma[w(|F_o|^4)]}\right)^{1/2} \text{ and } w=\frac{1}{(\sigma^2(I)+0.0004I^2)}$$

TABLE S4: Atomic coordinates ($x10^4$) and equivalent isotropic displacement parameters (Å^{2}x10^3) for $FAPbBr_3$ at 100 K (*Immm* space group) with estimated standard deviations in parentheses.

Label	x	y	z	Occupancy	U_{eq}*
Pb(1)	5000	7501(1)	7499(1)	1	12(1)
Br(1)	5000	5000	7773(2)	1	41(1)
Br(2)	7500	7500	7500	1	78(2)
Br(3)	5000	7247(3)	5000	1	53(1)
Br(4)	5000	7746(3)	10000	1	61(2)
Br(5)	5000	10000	7297(3)	1	83(2)
C(1)	2980(30)	10000	5000	1	86(4)
C(2)	2890(20)	5000	5000	1	87(4)
N(1)	2500(30)	10987(2)	5000	1	86(4)
N(2)	2410(20)	5000	4013(2)	1	87(4)

*U_{eq} is defined as one third of the trace of the orthogonalized U_{ij} tensor.

TABLE S5: Anisotropic displacement parameters (Å^{2}x10^3) for $FAPbBr_3$ at 100 K (*Immm* space group) with estimated standard deviations in parentheses.

Label	U_{11}	U_{22}	U_{33}	U_{12}	U_{13}	U_{23}
Pb(1)	9(1)	15(1)	13(1)	0	0	0(1)
Br(1)	66(2)	8(1)	49(2)	0	0	0
Br(2)	10(1)	106(3)	116(4)	-3(2)	-11(2)	-1(1)
Br(3)	91(2)	60(2)	7(1)	0	0	0
Br(4)	115(3)	53(2)	15(1)	0	0	0
Br(5)	170(5)	14(2)	65(2)	0	0	0

The anisotropic displacement factor exponent takes the form: $-2\pi 2[h^2a^{*2}U_{11} + ... + 2hka^*b^*U_{12}]$.

TABLE S6: Bond lengths (Å) for $FAPbBr_3$ at 100 K (*Immm* space group) with estimated standard deviations in parentheses.

Label	Distances (Å)
Pb(1)-Br(1)	2.9787(5)
Pb(1)-Br(2)	2.96058(15)
Pb(1)-Br(3)	2.9726(5)
Pb(1)-Br(4)	2.9741(5)
Pb(1)-Br(5)	2.9692(5)
C(1)-N(1)	1.300(18)
C(1)-N(2)	1.300(17)

TABLE S7: Bond angles (°) for $FAPbBr_3$ at 100 K (*Immm* space group) with estimated standard deviations in parentheses.

Label	Angles (°)
Pb(1)-Br(1)-Pb(1)'	167.51(9)
Pb(1)-Br(2)-Pb(1)'	180
Pb(1)-Br(3)-Pb(1)'	168.41(12)
Pb(1)-Br(4)-Pb(1)'	168.76(13)
Pb(1)-Br(5)-Pb(1)'	170.75(14)

S3. POLARIZATION-ORIENTATION (PO) RAMAN SCATTERING MEASUREMENTS

In polarization-orientation (PO) Raman scattering measurements, the crystal surface is excited by a linearly polarized laser (785 nm). The scattered light is then filtered by an analyzer for polarization parallel and perpendicular to the incident light. This measurement is repeated after rotating the polarization of the incident light by 5° (half-wave plate is rotated by 2.5°) while the sample position is fixed. The resulting false-color plots (fig. 2a in the main article and fig. S5) show the fluctuations in scattering intensity as a function of the angle between the polarization of the incident light and an arbitrary axis on the surface of the measured crystal. Figure S5 shows the temperature-dependent PO plots of $FAPbBr_3$ and $MAPbBr_3$ at 10 K, 80 K, and 300 K for both materials, and additionally at 150 K and 220 K for $FAPbBr_3$ only.

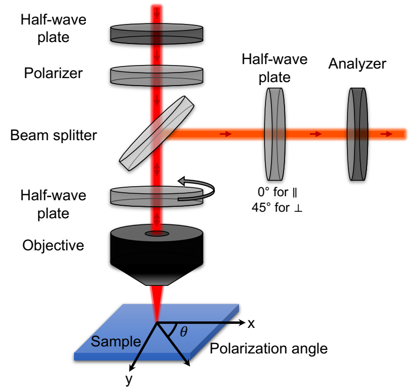

FIG. S4: Scheme of the polarization-orientation Raman scattering measurements, detailing the optical components in the setup.

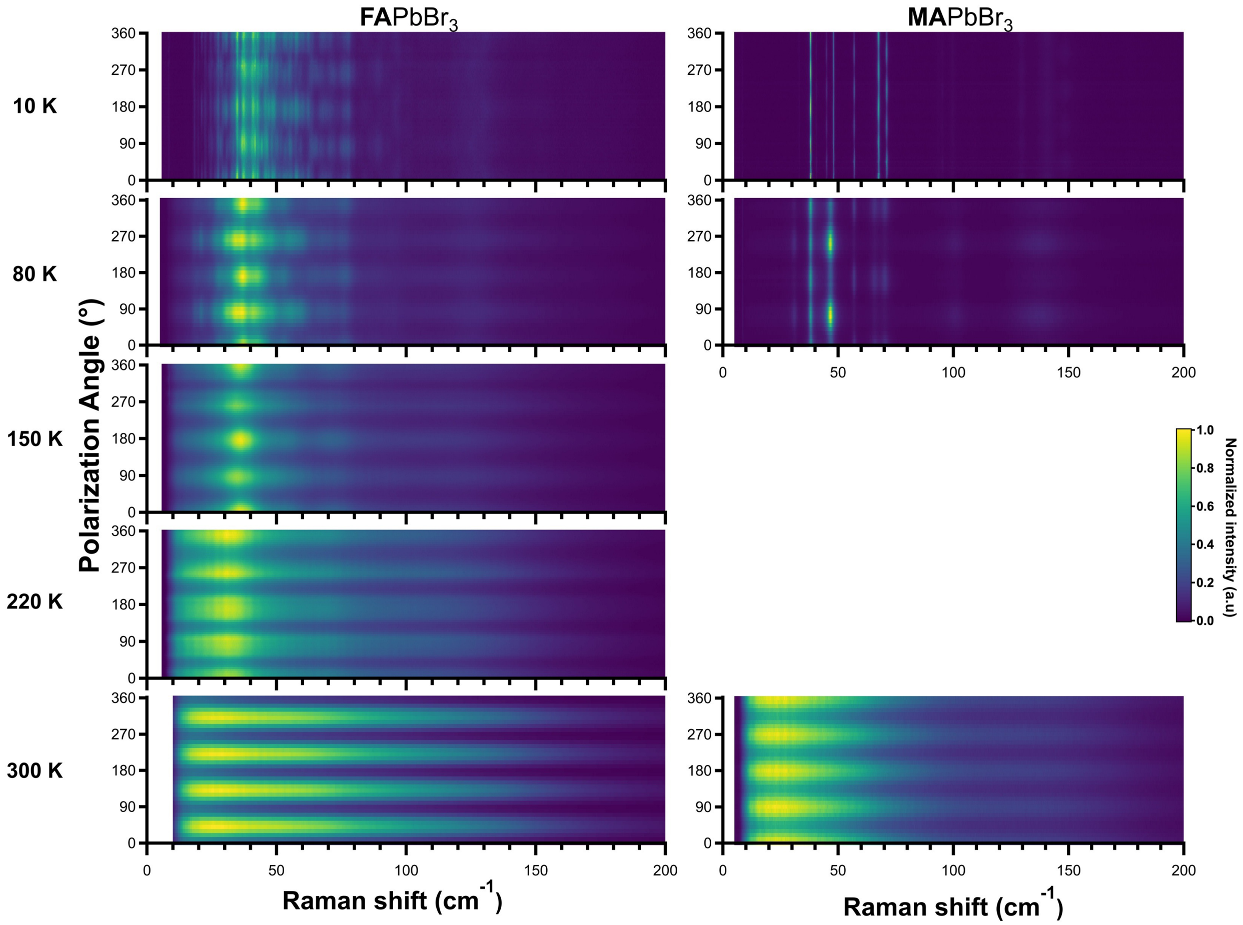

FIG. S5: Temperature-dependent, false-color polarization-orientation Raman plots of $FAPbBr_3$ and $MAPbBr_3$. All plots are presented for the parallel configuration. Intensities are separately normalized for each temperature, represented by the color scale.

S4. METHODS

S4.a. Raman scattering

Raman scattering measurements were conducted in a home-built back-scattering system[11,12] using a below band-gap 1.58 eV CW pump-diode laser (Toptica Inc., USA). The incident beam was linearly polarized by a Glan-laser polarizer (Thorlabs, USA), directed into a microscope (Zeiss, USA), and focused on the sample through a 0.55 NA/50x objective (Zeiss, USA). The excitation polarization was controlled by a zero-order half-wave plate (Thorlabs, USA) and was rotated in small increments (5°) between measurements. The back-scattered beam was collected by the objective and passed through another polarizer to collect only light that was scattered either parallel or perpendicular to the incident polarization. Rayleigh scattering was reduced by passing the beam through a volume holographic beam-splitter and two OD>4 notch filters (Ondax Inc., USA). Finally, the beam was focused on a 1 m long spectrometer (FHR 1000, Horiba) dispersed by 1800 gr/mm grating, achieving $\approx 0.3\ cm^{-1}$ spectral resolution, and detected by Si CCD (Horiba Inc., USA). For temperature control, all crystals were mounted into liquid Nitrogen- or liquid Helium-cooled optical cryostat (Janis Inc., USA). Unpolarized spectra were obtained by summing the spectra of all measured incident polarizations collected in parallel and perpendicular configurations and normalizing them to the maximum intensity. The temperature-dependent Raman results presented in the main text were reversible with temperature.

S4.b. First-principles calculations

The vibrational density of states of cubic $FAPbBr_3$ and $MAPbBr_3$ was calculated with the finite-displacement method, using density functional theory (DFT) and the phonopy-package[13]. The DFT calculations were done with the VASP package[14,15], employing the PBE functional[16], the "normal" version[17] of the projector-augmented wave (PAW) potentials[18] and a TS-scheme to correct for dispersive contributions[19]. The energy threshold was set to 10^{-8} eV, and a Γ-centered k-point grid of 4x4x4 k-points was used for the 2x2x2 supercells. The plane-wave cutoff was set to 700 eV for $FAPbBr_3$ and 800 eV for $MAPbBr_3$.

As mentioned in the main text, we computed the cubic phase of the crystals because we found the energetic landscape of orthorhombic (i.e., low-temperature phase) $FAPbBr_3$ to be

highly sensitive to small geometrical changes of the FA molecule and associated distortions of the inorganic lattice. The result of this is a considerably "corrugated" potential energy surface with many essentially isoenergetic local minima, which prevents us from obtaining a numerically stable, well-defined equilibrium structure of the $FAPbBr_3$ orthorhombic crystal that would be a prerequisite for subsequent phonon calculations. The orientation of the MA/FA molecules in the primitive cell was optimized by relaxing ionic and lattice degrees of freedom with the `GADGET` tool[20] until forces were below a threshold of 10^{-3} eV/Å. While the cubic symmetry was not imposed during the optimization of the structure, the optimized lattice did not strongly deviate from it: e.g., angles among lattice vectors were found to be 90 ± 3 degrees for $MAPbBr_3$ and 90 ± 7 degrees for $FAPbBr_3$, with lattice parameters of ∼5.95Å and ∼ 6.22Å, respectively. The phonon calculations proceeded these relaxations in a periodically-repeated 2x2x2 supercell, in which the molecule's orientation was kept fixed. Note that for the case of $FAPbBr_3$, we found a presence of several local minima which resulted in instabilities visible in the phonon dispersion relations as imaginary frequencies at Γ. Through consecutive rotations of the FA molecule along different directions, we could further lower the total energy and finally obtain phonon dispersion relations without the presence of imaginary modes at Γ, which was not possible for the orthorhombic case.

---

[1] M. I. Saidaminov, A. L. Abdelhady, G. Maculan, and O. M. Bakr, Retrograde solubility of formamidinium and methylammonium lead halide perovskites enabling rapid single crystal growth, Chem. Commun. **51**, 17658 (2015).

[2] D. R. Ceratti, Y. Rakita, L. Cremonesi, R. Tenne, V. Kalchenko, M. Elbaum, D. Oron, M. A. C. Potenza, G. Hodes, and D. Cahen, Self-healing inside APbBr3 halide perovskite crystals, Adv. Mater. **30**, 1706273 (2018).

[3] G. M. Sheldrick, *SHELXT* – Integrated space-group and crystal-structure determination, Acta. Crystallogr. A **71**, 3 (2015).

[4] G. M. Sheldrick, Crystal structure refinement with *SHELXL*, Acta. Crystallogr. C **71**, 3 (2015).

[5] C. J. Howard and H. T. Stokes, Group-theoretical analysis of octahedral tilting in perovskites, Acta. Crystallogr. B **54**, 782 (1998).

[6] K. S. Aleksandrov, The sequences of structural phase transitions in perovskites, Ferroelectrics

14, 801 (1976).

[7] P. M. Woodward, Octahedral tilting in perovskites. II. Structure stabilizing forces, Acta. Crystallogr. B **53**, 44 (1997).

[8] V. Petříček, M. Dušek, and L. Palatinus, Crystallographic computing system jana2006: General featurescrystallographic computing system jana2006: General features, Z. Kristallogr. Cryst. Mater. **229**, 345 (2014).

[9] V. Petříček, M. Dušek, and J. Plášil, Crystallographic computing system jana2006: solution and refinement of twinned structures, Zeitschrift für Kristallographie - Crystalline Materials **231**, 583 (2016).

[10] A. Poglitsch and D. Weber, Dynamic disorder in methylammoniumtrihalogenoplumbates (II) observed by millimeter-wave spectroscopy, J. Chem. Phys. **87**, 6373 (1987).

[11] M. Asher, D. Angerer, R. Korobko, Y. Diskin-Posner, D. A. Egger, and O. Yaffe, Anharmonic lattice vibrations in small-molecule organic semiconductors, Adv. Mater. **32**, 1908028 (2020).

[12] R. Sharma, M. Menahem, Z. Dai, L. Gao, T. M. Brenner, L. Yadgarov, J. Zhang, Y. Rakita, R. Korobko, I. Pinkas, *et al.*, Lattice mode symmetry analysis of the orthorhombic phase of methylammonium lead iodide using polarized raman, Phys. Rev. Mater. **4**, 051601 (2020).

[13] A. Togo and I. Tanaka, First principles phonon calculations in materials science, Scr. Mater. **108**, 1 (2015).

[14] G. Kresse and J. Furthmüller, Efficient iterative schemes for ab initio total-energy calculations using a plane-wave basis set, Phys. Rev. B **54**, 11169 (1996).

[15] G. Kresse and J. Furthmüller, Efficiency of ab-initio total energy calculations for metals and semiconductors using a plane-wave basis set, Comput. Mater. Sci. **6**, 15 (1996).

[16] J. P. Perdew, K. Burke, and M. Ernzerhof, Generalized gradient approximation made simple, Phys. Rev. Lett. **77**, 3865 (1996).

[17] Specifically, we have chosen the following PAW potentials:[21] H, C, N, Br, Pb_d.

[18] G. Kresse and D. Joubert, From ultrasoft pseudopotentials to the projector augmented-wave method, Phys. Rev. B **59**, 1758 (1999).

[19] A. Tkatchenko and M. Scheffler, Accurate molecular van der waals interactions from ground-state electron density and free-atom reference data, Phys. Rev. Lett. **102**, 073005 (2009).

[20] T. Bučko, J. Hafner, and J. G. Ángyán, Geometry optimization of periodic systems using internal coordinates, J. Chem. Phys. **122**, 124508 (2005).

[21] PAW potentials available in VASP package, https://www.vasp.at/wiki/index.php/Available_PAW_potentials#Recommended_potentials_for_DFT_calculations, accessed: 2022-12-29.
