# Supplementary material for: Static and Dynamic Disorder in Formamidinium Lead Bromide Single Crystals
Source: J Phys Chem Lett. 2023 Feb 1;14(5):1288–93. doi: 10.1021/acs.jpclett.2c03337 (PMC9923750; doi:10.1021/acs.jpclett.2c03337)
Supplement: Supplementary file 3 — jz2c03337_si_003.pdf [file jz2c03337_si_003.pdf]

jz-2022-03337b.R1

Name: Peer Review Information for "Static and Dynamic Disorder in Formamidinium Lead Bromide Single Crystals"

First Round of Reviewer Comments

Reviewer: 1

Comments to the Author

Overview: In this work Reuveni et al. used THz Raman scattering and other techniques on FAPbBr3 single crystal perovskites to investigate disorder. They found that due to the large size of FA compared to MA, the inorganic framework is distorted in case of FAPbBr3 and this results in additional disorder in FAPbBr3. At higher temperatures dynamic disorder takes over where FAPbBr3 and MAPbBr3 may exhibit similar features. They have backed up their results using first-principle calculations.

Recommendation: This is an excellent study and very well written hence I recommend publication almost as is. I have one minor comment only. I don't need to review the manuscript again I leave this minor comment to the authors and the editor.

Minor comment: I would like to note that co-existence of static and dynamic disorder at non-zero T is expected for any lattice. I understand that in case of FA the authors detect a larger static disorder. What is the contribution of zero point energy of phonons in this static disorder? It has been shown that in case of MAPI [Ledinsky] and other perovskites with mixed cations [Zeiske] the total disorder is a mixture of static and dynamic. Then the static part may be due to static distortions of the lattice as well as zero point motion of phonons [Zeiske]. It's been noted in the latter work that a single harmonic model is not sufficient to explain experimental data on disorder versus energy which also points towards anharmonicities. I would suggest the authors to elaborate on the role of zero point energy and cite these above mentioned studies as they are highly relevant.

[Ledinsky] Ledinsky, Martin, et al. "Temperature dependence of the urbach energy in lead iodide perovskites." The journal of physical chemistry letters 10.6 (2019): 1368-1373.

[Zeiske] Zeiske, Stefan, et al. "Static Disorder in Lead Halide Perovskites." The journal of physical chemistry letters 13.31 (2022): 7280-7285.

Reviewer: 2

Comments to the Author

This paper reports a combined experimental (Raman scattering, single-crystal XRD) and computational (DFT) study comparing $MAPbBr_3$ and $FAPbBr_3$ to elucidate differences in static and dynamics disorder in these two materials. The authors report that $FAPbBr_3$ appears to be somewhat unique in that its PbBr sublattice exhibits intrinsic local static disorder coexisting with a well-defined average crystal structure. In contrast, $MAPbBr_3$ at low temperature is known to not be disordered.

I am hesitant to recommend this paper for the Journal of Physical Chemistry Letters. The results are very interesting and clearly backed by state-of-the arts experiments. What I am missing to clearly establish the significance of this work are any kind of predictions either for how these "unique" structural features of $FAPbBr_3$ affect its optoelectronic properties or whether $FAPbBr_3$ is really that unique, i.e., whether what is learned from this material could be used to make a prediction for which other perovskites could feature similar properties.

In terms of detailed suggestions I am mostly going to comment on the computational part:

1. I confess that I am confused about the phonon calculations and their interpretation. I understand the authors' argument for performing these calculations on the cubic structures. On the other hand: Given that these crystals are highly anharmonic at high temperatures, why is a phonon density of states from finite differences representative of the actual vibrational DOS of the system? Furthermore, should the cubic structures not display imaginary modes? How are the authors dealing with those in the vibrational DOS?

2. The main text and the SI contain no information about how the structures were prepared for the phonon calculations. How are the MA/FA orientations in the 2x2x2 supercells chosen? Which lattice parameters were used? How were the structures relaxed for the phonon calculations? Was the overall cubic symmetry of these structures retained during relaxation? In my experience both $MAPbBr_3$ and $FAPbBr_3$ exhibit strong distortions away from the cubic parent phase when relaxed (which are computational artifacts). All of these questions seem highly non-trivial to me but the SI does not really give any details that would allow other researchers to reproduce the authors results. How sensitive are the vDOS shown in Figure 1 to these details?

3. I understand that Figure 1 does not necessarily suggest a comparison between panels a on the one hand, and b and c on the other. Nonetheless, can the authors comment on comparing experimental Raman spectra and calculated vDOS? I assume that the latter contain Raman-inactive modes too?

4. Finally, and as a bit of a repetition of my more general comment above: Being the devils advocate here but in a material class featuring so many stable members, can FAPbBr really be described as being unique? Maybe to put this in more constructive terms: Can the authors make any predictions for other perovskite compositions leading to similar properties?

5. I lied, I have another one, but only out of interest: The authors are using very high plane wave energy cutoffs. I assume this is necessary to get well-converged phonons but am surprised by how large they are (Which PAWs were used? Please report in the SI.). How much do the phonon frequencies change if this criterion is relaxed a bit?

Author's Response to Peer Review Comments:

A cover letter with the response to the reviewers' comments is attached.

Also attached is the revised manuscript and supporting information with highlighted text changes and additions.

December 13, 2022

To: Journal of Physical Chemistry Letters, Editor

'Static and Dynamic Disorder in Formamidinium Lead Bromide Single Crystals' by G. Reuveni *et al.*

Dear Editor,

On behalf of all the authors, I would like to thank you for your November 28 email informing us about the status of our submitted manuscript. We are grateful for the positive response and the helpful, constructive feedback by the two reviewers. Considering their very useful recommendations and addressing their pertinent questions has helped us to further improve the quality of our manuscript.

As requested, you will find a detailed, point-by-point response letter below, together with a list of the changes made in response. A version of the main text where these changes are highlighted is attached for your convenience. With these, we respectfully ask that you consider our revised manuscript for publication in *JPCL*. Thank you very much for your consideration.

Sincerely,

Omer Yaffe

Reviewer 1:

Comment: Overview: In this work Reuveni et al. used THz Raman scattering and other techniques on FAPbBr3 single crystal perovskites to investigate disorder. They found that due to the large size of FA compared to MA, the inorganic framework is distorted in case of FAPbBr3 and this results in additional disorder in FAPbBr3. At higher temperatures dynamic disorder takes over where FAPbBr3 and MAPbBr3 may exhibit similar features. They have backed up their results using first-principle calculations.

Recommendation: This is an excellent study and very well written hence I recommend publication almost as is. I have one minor comment only. I don't need to review the manuscript again I leave this minor comment to the authors and the editor.

Response: We thank the reviewer for their recommendation.

Minor comment: I would like to note that co-existence of static and dynamic disorder at non-zero T is expected for any lattice. I understand that in case of FA the authors detect a larger static disorder. What is the contribution of zero point energy of phonons in this static disorder? It has been shown that in case of MAPI [Ledinsky] and other perovskites with mixed cations [Zeiske] the total disorder is a mixture of static and dynamic. Then the static part may be due to static distortions of the lattice as well as zero point motion of phonons [Zeiske]. It's been noted in the latter work that a single harmonic model is not sufficient to explain experimental data on disorder versus energy which also points towards anharmonicities. I would suggest the authors to elaborate on the role of zero point energy and cite these above mentioned studies as they are highly relevant.

[Ledinsky] Ledinsky, Martin, et al. "Temperature dependence of the urbach energy in lead iodide perovskites." The journal of physical chemistry letters 10.6 (2019): 1368-1373. [Zeiske] Zeiske, Stefan, et al. "Static Disorder in Lead Halide Perovskites." The journal of physical chemistry letters 13.31 (2022): 7280-7285.

Response: We thank the reviewer for reminding us about these important studies. Our revised version of the manuscript now discusses and cites these relevant studies.

Changes:

Added on p. 3:

... which adds to recent discussions on the pertinent mechanisms underlying the interplay of static and dynamic disorder in lead-halide perovskites.

Reviewer 2:

Comment:

This paper reports a combined experimental (Raman scattering, single-crystal XRD) and computational (DFT) study comparing $MAPbBr_3$ and $FAPbBr_3$ to elucidate differences in static and dynamics disorder in these two materials. The authors report that $FAPbBr_3$ appears to be somewhat unique in that its PbBr sublattice exhibits intrinsic local static disorder coexisting with a well-defined average crystal structure. In contrast, $MAPbBr_3$ at low temperature is known to not be disordered.

I am hesitant to recommend this paper for the Journal of Physical Chemistry Letters. The results are very interesting and clearly backed by state-of-the arts experiments. What I am missing to clearly establish the significance of this work are any kind of predictions either for how these "unique" structural features of $FAPbBr_3$ affect its optoelectronic properties or whether $FAPbBr_3$ is really that unique, i.e., whether what is learned from this material could be used to make a prediction for which other perovskites could feature similar properties.

Response: We thank the reviewer for their careful comments and validation. In response, we would like to stress that we emphasized in the abstract of the original article why the FA-based system is "unique", namely "*... because its inorganic sub-lattice exhibits intrinsic local static disorder that co-exists with a well-defined average crystal structure.*. The reviewer asks us the interesting question, whether this unique aspect would impact the optoelectronic properties or whether other perovskites may show similar behavior. We do not have any data or evidence at the moment to suggest that this would indeed be the case. However, our work has demonstrated that the origin of the static disorder is the inorganic sublattice, which makes up the electronic states close to the valence and conduction bands. Hence, the findings imply that these states could be impacted by static disorder. Furthermore, we have hypothesized that the FA-molecule is corrugating the inorganic sublattice, which would imply that similar effects could happen in other FA-based materials as well. We have elaborated on these issues in the revised version. Lastly, we changed the descriptive term "unique" to different wording.

Changes:

Abstract:

We show that formamidinium-based are distinct from methylammonium-based halide perovskite crystals because ...

Added on p. 5:

The notion is relevant for mechanistic understandings of the optoelectronic properties in FA-based lead-halide perovskites, since the electronic states due to the $PbBr_6$ framework are close to the band edges. In light of this and the improved stability and self-healing properties of FA-based materials ...

Added on p. 6:

Changed from *"this unique perovskite"* to *"this remarkable perovskite and guiding future studies of related FA-based perovskites."*

Comment: 1. I confess that I am confused about the phonon calculations and their interpretation. I understand the authors' argument for performing these calculations on the cubic structures. On the other hand: Given that these crystals are highly anharmonic at high temperatures, why is a phonon density of states from finite differences representative of the actual vibrational DOS of the system? Furthermore, should the cubic structures not display imaginary modes? How are the authors dealing with those in the vibrational DOS?

Response: The reviewer correctly pointed out that the system is anharmonic at high temperatures. If the purpose of the calculations would have been to quantitatively trace certain vibrational features at higher temperatures, we would indeed need to account for such effects. However, as stated in the original manuscript the goal of the calculations was to investigate the origin of the low-frequency components in the spectrum that result in significant static disorder for the FA-based system. Notably, this static disorder is already present at 10 K in absence of strong anharmonicity. Furthermore, the vibrational contributions of the inorganic sublattice dominating the static disorder a priori cannot be expected to change strongly as anharmonicity is becoming increasingly relevant at higher temperatures. On the contrary, as shown for the case of $MAPbBr_3$ in [J. Phys. Chem. C 121, 3724 (2017)], the atomic contributions to the vDOS are stable throughout wide ranges of temperature. We have modified the main text to clarify this valid point.

Moreover, the reviewer's expectation of imaginary modes in the cubic structures is correct. Our focus was on the low-frequency region because we are investigating what causes the unusual Raman spectrum in that regime. The imaginary features mentioned by the reviewer have been studied extensively in the literature, which is why we did not show and further discuss them in our work. For the same reasons, we also did not show or discuss the high-frequency data associated with the molecular vibrations. We

agree that it would be useful for the readability of the manuscript to mention that these other features exist and clarify that we did not show them in the vDOS.

Changes:

Added on p. 4:

We note in passing that the identification of the origin of the static disorder in FAPbBr$_3$ by harmonic phonon calculations is not expected to be modified by anharmonic effects, which in these materials do not strongly alter atomic contributions to the vDOS.

Added on p. 2, caption of Fig. 1:

Note that the vDOS also includes contributions from Raman-inactive modes, and that it is shown for a selected frequency region which does not, e.g., show contributions from imaginary modes and higher-frequency molecular modes.

Comment: 2. The main text and the SI contain no information about how the structures were prepared for the phonon calculations. How are the MA/FA orientations in the 2x2x2 supercells chosen? Which lattice parameters were used? How were the structures relaxed for the phonon calculations? Was the overall cubic symmetry of these structures retained during relaxation? In my experience both MAPbBr$_3$ and FAPbBr$_3$ exhibit strong distortions away from the cubic parent phase when relaxed (which are computational artifacts). All of these questions seem highly non-trivial to me but the SI does not really give any details that would allow other researchers to reproduce the authors results. How sensitive are the vDOS shown in Figure 1 to these details?

Response: We agree with the reviewer that these details are important and have now supplied them in the revised version of the SI, addressing all questions by the reviewer and also providing additional information.

Regarding the question of sensitivity, we never found any indication in any of our data that our main message - the inorganic sublattice determines static disorder in FAPbBr$_3$ - would be modified.

Changes:

Added on p. S12/13:

The orientation of the MA/FA molecules in the primitive cell was optimized by relaxing ionic and lattice degrees of freedom with the ***GADGET*** *tool until forces were below a threshold of 10^{-3} eV/Å. While the cubic symmetry was not imposed during the optimization of the structure, the optimized lattice did not strongly deviate from it: e.g., angles among lattice vectors were found to be 90 $\pm$ 3 degrees for MAPbBr$_3$ and 90 $\pm$ 7 degrees for FAPbBr$_3$, with lattice parameters of ∼5.95Å and ∼ 6.22Å, respectively. The phonon calculations proceeded these relaxations in a periodically-repeated 2x2x2 supercell, in which the orientation of the molecule was kept fixed. Note that for the case of FAPbBr$_3$, we found a presence of several local minima which resulted in instabilities visible in the phonon dispersion relations as imaginary frequencies at Γ. Through consecutive rotations of the FA molecule along different directions we could further lower the total energy and finally obtain a phonon dispersion relations without a presence of imaginary modes at Γ, which was not possible for the orthorhombic case.*

Comment: 3. I understand that Figure 1 does not necessarily suggest a comparison between panels a on the one hand, and b and c on the other. Nonetheless, can the authors comment on comparing experimental Raman spectra and calculated vDOS? I assume that the latter contain Raman-inactive modes too?

Response: The reviewer is correct that the vDOS includes contributions from all modes at all $\mathbf{q}$

points, while the Raman effect only probes Raman-active modes at $\mathbf{q} = \Gamma$. We are happy to add an analogous clarification to the main text.

Changes:

See response to the 2nd comment.

Comment: 4. Finally, and as a bit of a repetition of my more general comment above: Being the devils advocate here but in a material class featuring so many stable members, can FAPbBr really be described as being unique? Maybe to put this in more constructive terms: Can the authors make any predictions for other perovskite compositions leading to similar properties?

Response: Please see our detailed response to the general comment by the reviewer. In short, we have clarified the meaning of what is unique for this system, elaborated on further potential implications for other compounds and properties, and revised the wording in regard to the term "unique".

Changes:

Comment: 5. I lied, I have another one, but only out of interest: The authors are using very high plane wave energy cutoffs. I assume this is necessary to get well-converged phonons but am surprised by how large they are (Which PAWs were used? Please report in the SI.). How much do the phonon frequencies change if this criterion is relaxed a bit?

Response: We have converged the energy cutoff with respect to all reported quantities. Unfortunately, we are not entirely certain what the reviewer is alluding to when mentioning "relaxing a bit". If the energy cutoff is meant, the answer will also depend on whether one recomputes lattice constants or not. Most importantly, our main insight about the assignment of the modes in the low-frequency regions does not depend on refined numerical details of the calculations, which are, however, important for reporting converged data. Beyond this, we agree that it might be useful to mention the version of the PAW potentials, which we have added to the revised SI.

Changes:

Added on p. S12:

... the "normal" version of the PAW pseudopotentials ...

Further changes:

- Slightly changed the wording on page 2, left column, to enhance readability.
- SI reference was removed. Direct referencing to the SI was added on p. 5.

jz-2022-03337b.R2

Name: Peer Review Information for "Static and Dynamic Disorder in Formamidinium Lead Bromide Single Crystals"

Second Round of Reviewer Comments

Reviewer: 2

Comments to the Author

I thank the authors for answering my questions. I am happy with the revised version of the manuscript. A fundamental understanding of the origin, character, and temperature-dependence of static and dynamic order in halide perovskites as provided by this paper is a crucial prerequisite for advances in material design for optoelectronic applications and beyond. The significance of this work therefore goes beyond the study of two specific materials and so I think publication in the Journal of Physical Chemistry Letters is well justified.

I have one remaining minor comment, regarding my previous question about the PAWs. The authors write: ”We have converged the energy cutoff with respect to all reported quantities. Unfortunately, we are not entirely certain what the reviewer is alluding to when mentioning “relaxing a bit”. If the energy cutoff is meant, the answer will also depend on whether one recomputes lattice constants or not.

Most importantly, our main insight about the assignment of the modes in the low-frequency regions does not depend on refined numerical details of the calculations, which are, however, important for reporting converged data. Beyond this, we agree that it might be useful to mention the version of the PAW potentials, which we have added to the revised SI.”

I was previously referring to the convergence of the phonon frequencies with respect to the energy cutoff. I agree with you that the result would depend on whether lattice constants were re-computed (they should be if one were to test this), but was hoping for a more concrete answer. Just to be clear: I have no doubts that your results are converged – I was wondering whether you had specific reasons for using a particularly stringent energy cutoff? My question about the PAWs was also inspired by this train of thought, but I should have been clearer: Which electronic configurations were used? I guess I am not sure what the “normal” PAWs are, but if you are referring to the ones that do not include semicore electrons, I still do not have a good answer for why such a large energy cutoff is necessary. Why do I want to know this even though it clearly does not change the main results of the study? Just to ensure that others who might want to reproduce these results understand the error bars (presumably tiny) of

such a relatively time- and resource-heavy calculation and to promote the responsible use of computational resources in the field.

I think the information about the electronic configuration of the pseudopotentials should be added to the SI for reasons of reproducibility. I leave it to the authors whether they want to add more information about the convergence of the phonons with respect to the energy cutoff.

Author's Response to Peer Review Comments:

A response letter with details on SI changes is attached.

January 1, 2023

To: Journal of Physical Chemistry Letters, Editor

'Static and Dynamic Disorder in Formamidinium Lead Bromide Single Crystals' by G. Reuveni *et al.*

Dear Editor,

On behalf of all the authors, I would like to thank you for your December 28 email informing us about the status of our submitted manuscript. We are grateful for the positive response and recommendation by the reviewer. Their remaining minor question has been clarified.

As requested, you will find a response letter below, together with the one minor change made in response. Because this only added one footnote and one reference to the SI, we attach it together with the previously submitted version of the main without any highlights. With these, we believe our revised manuscript is ready for publication in *JPCL*. Thank you very much for your consideration.

Sincerely,

Omer Yaffe

Reviewer 2:

Comment: I thank the authors for answering my questions. I am happy with the revised version of the manuscript. A fundamental understanding of the origin, character, and temperature-dependence of static and dynamic order in halide perovskites as provided by this paper is a crucial prerequisite for advances in material design for optoelectronic applications and beyond. The significance of this work therefore goes beyond the study of two specific materials and so I think publication in the Journal of Physical Chemistry Letters is well justified.

I have one remaining minor comment, regarding my previous question about the PAWs. The authors write: "We have converged the energy cutoff with respect to all reported quantities. Unfortunately, we are not entirely certain what the reviewer is alluding to when mentioning "relaxing a bit". If the energy cutoff is meant, the answer will also depend on whether one recomputes lattice constants or not. Most importantly, our main insight about the assignment of the modes in the low-frequency regions does not depend on refined numerical details of the calculations, which are, however, important for reporting converged data. Beyond this, we agree that it might be useful to mention the version of the PAW potentials, which we have added to the revised SI."

I was previously referring to the convergence of the phonon frequencies with respect to the energy cutoff. I agree with you that the result would depend on whether lattice constants were re-computed (they should be if one were to test this), but was hoping for a more concrete answer. Just to be clear: I have no doubts that your results are converged – I was wondering whether you had specific reasons for using a particularly stringent energy cutoff? My question about the PAWs was also inspired by this train of thought, but I should have been clearer: Which electronic configurations were used? I guess I am not sure what the "normal" PAWs are, but if you are referring to the ones that do not include semicore electrons, I still do not have a good answer for why such a large energy cutoff is necessary. Why do I want to know this even though it clearly does not change the main results of the study? Just to ensure that others who might want to reproduce these results understand the error bars (presumably tiny) of such a relatively time- and resource-heavy calculation and to promote the responsible use of computational resources in the field.

I think the information about the electronic configuration of the pseudopotentials should be added to the SI for reasons of reproducibility. I leave it to the authors whether they want to add more information about the convergence of the phonons with respect to the energy cutoff.

Response: We thank the reviewer for their recommendation and clarifying their question. There are "soft" and "hard" PAW potentials in addition to the "normal" ones that we have used. We agree that it could be helpful to list the exact PAW potentials employed in our study and are happy to provide this information to the revised version of the SI.

Changes:

Added a footnote on p. S12, listed as endnote 17 in the SI, which says:

Specifically, we have chosen the following PAW potentials:[21] H, C, N, Br, Pb_d

citing the following reference:

[21] PAW potentials available in VASP package, `https://www.vasp.at/wiki/index.php/Available_PAW_potentials#Recommended_potentials_for_DFT_calculations`, accessed: 2022-12-29.
